# A case control study on psychiatric disorders in Hashimoto disease and euthyroid goitre: not only depressive but also anxiety disorders are associated with thyroid autoimmunity

**DOI:** 10.1186/1745-0179-1-23

**Published:** 2005-11-10

**Authors:** Mauro Giovanni Carta, Maria Carolina Hardoy, Bernardo Carpiniello, Andrea Murru, Anna Rita Marci, Fiora Carbone, Luca Deiana, Mariangela Cadeddu, Stefano Mariotti

**Affiliations:** 1 Division of Psychiatry, Department of Public Health, University of Cagliari, Italy; 2 Department of Internal Medicine, University of Cagliari, Italy

**Keywords:** Autoimmunity, Anxiety disorders, Goitre Hashimoto disease, Mood disorders, Thyroid antibodies

## Abstract

**Objective:**

To evaluate the association between mood and anxiety disorders in Hashimoto disease and Euthyroid Goitre in a case control study.

**Methods:**

Cases included 19 subjects with Hashimoto disease in euthyroid phase, 19 subjects with euthyroid goitre, 2 control groups each of 76 subjects matched (4/1) according to age and sex drawn from the data base of a community based sample. Psychiatric diagnoses were formulated using the International Composite Diagnostic Interview Simplified, according to DSM-IV criteria. All subjects underwent a complete thyroid evaluation including physical examination, thyroid echography and measure of serum free T4 (FT4), free T3 (FT3), thyroid-stimulating hormone (TSH) and anti-thyroid peroxidase autoantibodies (anti-TPO). Results: Subjects with Hashimoto disease showed higher frequencies of lifetime Depressive Episode (OR = 6.6, C.L. 95% 1.2–25.7), Generalized Anxiety Disorders (OR = 4,9 Cl 95% 1.5–25.4) and Social Phobia (OR = 20.0, CL 95% 2.3–153.3) whilst no differences were found between subjects with goitre and controls.

**Conclusion:**

The study seems to confirm that risk for depressive disorders in subjects with thyroiditis is independent of the thyroid function detected by routine tests and indicates that not only mood but also anxiety disorders may be associated with Hashimoto disease.

## Introduction

Clinical and epidemiological studies seem to indicate that an association between high levels of thyroid autoantibodies may be implicated in the increased frequencies of mood disorders observed in thyroid disease, independently of impairment of thyroid function [1-3]. A study carried out by our group suggested a possible role of thyroid autoimmunity in the association between celiac disease and panic disorder and major depressive disorder [4].

The aim of the present study was to compare 2 clinical samples of subjects, one with Hashimoto disease (in euthyroid phase) and the second with euthyroid goitre versus controls drown from a community sample, in order to clarify whether the association of mood and anxiety disorder in Hashimoto disease is evident prior to impairment of thyroid dysfunction.

## Methods

### Design: case control study

Cases: 19 subjects, 18 females, mean age 39.7+/-12.6, with Hashimoto disease in euthyroid phase and 19 subjects, 16 females, mean age 38.1+/-10.4, with euthyroid goitre, all of whom were attending the Endocrinological Unit of the Department of Internal Medicine, University of Cagliari, Italy.

Two control groups, each of 76 subjects aged 18–64 years, were obtained by matching each "case" with four "controls" randomly selected on the basis of demographic characteristics (sex and age) from the data base of a larger population enrolled during a previous epidemiological study aimed at defining the prevalence of psychiatric [5] and thyroid diseases [6] in Sardinia. Controls were selected by a technique of randomisation after matching. For each case, a cell was formed containing all possible sex and age-matched controls (~1.0 year). The number of cells varied from 5 to 12 controls; a control was extracted at random from each cell. Exclusion criteria for control groups were the presence of abnormal values of serum free T4, free T3, thyroid-stimulating hormone (TSH) and anti-thyroid peroxidase autoantibodies (anti-TPO) as will be specified.

Two standardized forms were used to acquire information concerning: demographic data, state of health and use of social and health services. Psychiatric diagnosis was made using the Italian Simplified version of the Composite International Diagnostic Interview (CIDIS) [7]. The computer elaboration of data obtained enabled prevalence of psychiatric disorders according to DSM-IV [8] diagnostic criteria to be calculated.

All subjects underwent a complete thyroid evaluation including physical examination, thyroid echography and measure of serum free T4 (FT4), free T3 (FT3), thyroid-stimulating hormone (TSH) and anti-thyroid peroxidase autoantibodies (anti-TPO). FT4 and FT3 were measured by means of a chromatographic method based on separation of free T4 on Lisophase columns (Technogenetics, Milan, Italy; normal values: FT4 6.6–16 pg/ml; FT3 2.8–5.6 pg/ml); TSH was measured by a chemiluminescent method (Ortho-Clinical Diagnostics Amersham, U.K.) with normal values ranging from 0.3–3.0 μU/ml. Thyroid echography was performed using a "real time" echograph (ALOKA Mod SSD 500 with a small parts 7.5 Mhz sound. Anti-TPO, considered as the most sensitive and specific marker of thyroid autoimmunity [9] was determined by RIA (Sorin Biomedica Diagnostics, Saluggia, Italy) with a cut-off value of 20 IU/ml.

## Results

Subjects with Hashimoto disease displayed high frequencies of lifetime Depressive Episodes (OR = 6.6, C.L. 95% 1.2–25.7), Generalized Anxiety Disorders (OR = 4,9 Cl 95% 1.5–25.4), Social Phobia (OR = 20.0, CL 95% 2.3–153.3) and Primary Sleep Disorders (OR = 20.0, CL 95% 2.3–153.3); a tendency towards an increased frequency of Panic Disorder was observed, although statistical significance was not reached (OR = 1.8, 0.1–24.6). No differences were found in the evaluation of lifetime prevalence of DSM-IV Psychiatric Disorders between patients with Euthyroid goitre and controls (Table 1).

## Discussion

This study seems to indicate an association between the presence of a lifetime diagnosis of mood or anxiety disorder and Hashimoto disease without functional thyroid impairment. No difference was observed in mood and anxiety disorders between goitre cases and controls.

These findings are congruent with the results of a previous study [10] that indicated a risk for Depressive episode in subjects with antiTPO+ in a general population sample without selection by medical or psychiatric health facilities, and are consistent with several previous clinical studies providing evidence of a significant association of mood disorders or post-partum depression and symptomless autoimmune thyroiditis with or without sub-clinical hypothyroidism [11].

The above-cited community survey found that subjects with at least one lifetime diagnosis of anxiety disorders presented anti-TPO+ more frequently than subjects without mood or anxiety disorders. However, no specific anxiety diagnosis was found to be associated with anti-TPO+, although General Anxiety Disorder showed a strong trend toward association (P < 0.058). Anxiety Disorder Not Otherwise Specified was more frequently observed with anti-TPO+, but this is only a sub-threshold condition. A previous research carried out on celiac patients indicated thyroid autoimmunity as possible risk factor for panic disorder [3]. In the present study, the latter diagnosis was shown to be more frequent between Hashimoto disease and controls, although statistical significance was not reached.

Indeed, these 3 surveys seem to indicate a congruent trend of possible risk for anxiety disorders such Generalized Anxiety Disorder, Social Phobia and Panic Disorder in Hashimoto Disease.

To our knowledge, the data concerning the association between sleep disorders and Hashimoto disease is the first such evidence published in literature, although it may be consistent with a dysregulation of sleep architecture in subjects with sub-clinical thyroid impairment [12].

A sub-clinical dysfunction of axis thyrotropin releasing hormone (TRH) thyroid stimulating hormone (TSH) with consequent alteration of circadian rhythms of TSH has been hypothesized in several depressive disorders. Indeed, this hypothesis may explain why some forms of mood disorders were associated with anti-TPO+ without hypothyroidism, as defined by blood routine tests. A slight reduction in thyroid hormone secretion such as that found in sub-clinical hypothyroidism may affect cognition and mood [13]. At variance with other tissues which mainly rely on peripherally generated triiodothyronine, the brain utilizes preferentially circulating thyroxine directly secreted by the thyroid gland and may become hypothyroid before other organs [10].

Alternatively, autoimmunity may be implicated in some form of extra-thyroid disease associated (even indirectly) with the depressive symptomatology.

In Hashimoto disease the onset of vasculitis is frequently observed and would seem to be directly correlated to the duration of the illness [14].

In this disease brain perfusion modifications would appear to be of an aspecific nature; recently however, Spect examination revealed a marked compromising of the left cingulum posterioris, thus related in the etiology of associated affective disorders [14].

Recent evidence suggests that thyroid autoimmunity may be affected by the Hypothalamic-pituitary-adrenal axis (HPA) through the balance of proinflammatory and antiinflammatory cytokines [15,16]. In line with this view, the increased frequency of post-partum depression, associated to the fact that pregnancy would seem to be a "protected" period, could explain at least in part the consequences on thyroid autoimmunity elicited by HPA-related modifications to the immunitary axis. Indeed, similar phenomena are observed in both rheumatoid arthritis and multiple sclerosis and therefore Hashimoto vasculitis may also be involved [17].

### Limitations

The potential of this study is limited by the small sample size, particularly regard to psychiatric diagnoses less frequently observed in the general population, such as Panic Disorder; the extension of the findings is therefore rather limited.

## Conclusion

This study seems to indicate an association between the presence of a lifetime diagnosis of mood or anxiety disorder and sleep disturbance and anti-TPO+. The findings obtained suggest the need for further longitudinal studies aimed at clarifying the association between anxiety and depressive disorders and anti-TPO+.
